# MSFSP: A Novel miRNA–Disease Association Prediction Model by Federating Multiple-Similarities Fusion and Space Projection

**DOI:** 10.3389/fgene.2020.00389

**Published:** 2020-04-30

**Authors:** Yi Zhang, Min Chen, Xiaohui Cheng, Hanyan Wei

**Affiliations:** ^1^School of Information Science and Engineering, Guilin University of Technology, Guilin, China; ^2^School of Computer Science and Technology, Hunan Institute of Technology, Hengyang, China; ^3^School of Pharmacy, Guilin Medical University, Guilin, China

**Keywords:** disease similarity, miRNA similarity, multiple-similarities fusion, space projection, computational prediction model

## Abstract

Growing evidences have indicated that microRNAs (miRNAs) play a significant role relating to many important bioprocesses; their mutations and disorders will cause the occurrence of various complex diseases. The prediction of miRNAs associated with underlying diseases *via* computational approaches is beneficial to identify biomarkers and discover specific medicine, which can greatly reduce the cost of diagnosis, cure, prognosis, and prevention of human diseases. However, how to further achieve a more reliable prediction of potential miRNA–disease associations with effective integration of different biological data is a challenge for researchers. In this study, we proposed a computational model by using a federated method of combined multiple-similarities fusion and space projection (MSFSP). MSFSP firstly fused the integrated disease similarity (composed of disease semantic similarity, disease functional similarity, and disease Hamming similarity) with the integrated miRNA similarity (composed of miRNA functional similarity, miRNA sequence similarity, and miRNA Hamming similarity). Secondly, it constructed the weighted network of miRNA–disease associations from the experimentally verified Boolean network of miRNA–disease associations by using similarity networks. Finally, it calculated the prediction results by weighting miRNA space projection scores and the disease space projection scores. Leave-one-out cross-validation demonstrated that MSFSP has the distinguished predictive accuracy with area under the receiver operating characteristics curve (AUC) of 0.9613 better than that of five other existing models. In case studies, the predictive ability of MSFSP was further confirmed as 96 and 98% of the top 50 predictions for prostatic neoplasms and lung neoplasms were successfully validated by experimental evidences and supporting experimental evidences were also found for 100% of the top 50 predictions for isolated diseases.

## Introduction

The microRNAs (miRNAs) widely found in eukaryotes are those non-coding RNAs of about 20–25 nucleotides (Iorio et al., 2005). Life processes such as cell growth (Fernando et al., 2012; Zhu et al., 2016), differentiation (Miska, 2005), proliferation (Cheng et al., 2005), aging (Xu et al., 2004), signal transduction (Carthew and Sontheimer, 2009), etc. have been found to be associated with miRNAs. Increasing evidences continually confirm that complex diseases in humans including cancers, Alzheimer, diabetes, and lymphoma are closely related to miRNAs. In addition, some former researches proved that miRNAs can be considered as tumor genes or tumor suppressor genes. Therefore, inferring novel miRNA–disease associations have clinical significance for various human diseases due to miRNAs' potential roles in diagnosis biomarkers and treatment targets. Massive associations have been obtained *via* traditional biotic experiments and stored in some public databases. The traditional bio-experimental methods have high precision, but whose process is complex and time-consuming (Liang et al., 2019). Predicting and ranking potential miRNA–disease associations effectively and rapidly *via* computational identification methods are extremely vital to speed up the bio-experimental validation processes as well as reduce the blindness and time consumption of bio-experiments (Chen et al., 2015c, 2019c; Zeng et al., 2016b; Peng et al., 2017a, 2020).

On the basic assumption that functionally related miRNAs tend to be associated with phenotypically similar diseases and *vice versa* (Lu et al., 2008; Bandyopadhyay et al., 2010; Wang et al., 2010), various computational identification methods have been proposed continuously (Chen et al., 2017d, 2018c; Chen and Qu, 2018). Jiang et al. (2010a) proposed a miRNA–disease association prediction model that first used the hypergeometric distribution and constructed the functionally related miRNA network through the number of shared target genes to uncover the associations between miRNAs and diseases, but it needs to integrate other bioinformatics sources to improve model performance. Jiang et al. (2010b) proposed an approach that prioritized disease-related miRNAs based on integrating genomic data. Li et al. (2011) proposed a computational framework with which to prioritize human cancer-related miRNAs; it used the functional consistency score of miRNA-target genes and cancer-related genes to measure the associations between cancer and miRNAs. Xu et al. (2014) systematically prioritized disease-specific miRNAs by using the known disease genes and context-dependent miRNA-target interactions derived from the expression data of a matched miRNA–miRNA pair. Lack of excellent predictive performance of the above-mentioned methods may be attributed to the high false positive rate of the target genes.

Li J. et al. (2014) utilized recommendation systems to predict the associations between environmental factors, miRNAs, and diseases, but these cannot predict isolated diseases (without any known associated miRNAs) and new miRNAs (without any known associated diseases). Zhang Y. et al. (2019) used bipartite network projection (LSGSP) with known associations to reconstruct the family information, miRNA similarity network, and disease similarity network for predicting the potential miRNA–disease associations. Although LSGSP does not need negative samples, it cannot achieve good performance only with limited number of known associations. Chen et al. (2018h) proposed a bipartite recommendation algorithm to predict miRNA–disease associations (BNPMDA) that improved the prediction accuracy distinctly with the utilization of bias ratings. Chen et al. (2018b) proposed a novel information diffusion method based on network consistency (IDNC) for uncovering disease-related miRNAs. Despite not needing negative samples and simple algorithm design, too many parameters in different databases make IDNC take a long time to find the optimal values.

In recent years, some researchers have attempted to use the topological similarity of graph to predict a miRNA–disease association (Nalluri et al., 2015; Chen et al., 2016b, 2017c, 2018e; Sun et al., 2016; You et al., 2017; Zeng et al., 2018). Chen et al. (2017b) proposed the super-disease and miRNA concepts to design a novel computational model with which to infer miRNA–disease associations. Bipartite heterogeneous network method based on co-neighbor (Chen et al., 2019a), ELLPMDA of ensemble learning and link prediction (Chen et al., 2018j), and label propagation model with linear neighborhood (Li et al., 2018) were used for various types of miRNA–disease association prediction, but those did not figure out the easy way for parameter optimization. Random walk on heterogeneous network (Chen et al., 2012, 2016a, 2018a; Xuan et al., 2015; Liu et al., 2017; Luo and Xiao, 2017; Mugunga et al., 2017; Peng et al., 2018) used for inferring miRNA–disease associations has achieved excellent prediction results with global attributes, but all of their results were partial to such miRNAs that have more known associations with diseases.

Inspired by the successful application of machine learning methods in the field of bioinformatics, many researchers used supervised machine learning methods to predict a miRNA–disease association (Chen et al., 2015a,b, 2017a, 2018d,f, 2019a,b,d; Luo et al., 2017a; Xuan et al., 2018, 2019b; Wang C.-C. et al., 2019; Wang L. et al., 2019; Zhang L. et al., 2019; Zhao et al., 2019), but which need negative samples for training. Because it is hard to obtain the experimentally verified less-known miRNA–disease associations and negative samples, some semi-supervised learning approaches (such as regularized least squares) with remarkable prediction results were proposed (Chen and Huang, 2017; Chen et al., 2017c, 2018k; Peng et al., 2017b; Xu et al., 2019). Chen and Huang (2017) used Laplacian regularized sparse subspace learning for miRNA–disease association prediction (LRSSLMDA); it projected diverse statistical feature profiles into a common subspace and selected important diverse features with a L1-norm constraint. Jiang et al. (2018) proposed a novel similarity kernel fusion (SKF) method that integrated multiple-similarity kernels to construct the accurate network similarity on which to utilize Laplacian regularized least squares for potential associations inference. It can avoid to lose the initial information during the process and can eliminate some noises in integrated similarity kernels. Luo et al. (2017b) presented a semi-supervised method with Kronecker regularized least squares to predict the potential (or missing) miRNA–disease associations. However, the above semi-supervised solutions without the need for negative samples still have the limitation in initial values setting and optimal parameters of iteration selecting. Zeng et al. (2016a), Li et al. (2017), Chen et al. (2018g), Xiao et al. (2018), Xuan et al. (2019a), Xuan et al. (2019c), and Peng et al. (2017b) utilized the matrix completion to infer the potential miRNA–disease associations. Chen et al. (2018i) uncovered the potential miRNA–disease associations through integrating low-rank matrix decomposition and the sparse learning method. Qu et al. (2019) utilized matrix decomposition and label propagation to infer potential miRNA–disease associations. Tang et al. (2019) made full use of the miRNA functional similarity, the disease semantic similarity, and a dual Laplacian regularization term to work for the matrix completion of miRNA–disease associations. Chen et al. (2020) proposed a new computational model (NCMCMDA) that innovatively integrated neighborhood constraint with matrix completion to find out the absent miRNA–disease associations. Even though all of the above methods only needed experimentally validated miRNA–disease associations to make prediction with a good prediction effect, the optimal parameters selection still cannot be solved very well. Although computational prediction models have attracted a lot of interests in recent years and many distinguished research progresses have been achieved, the identification of the potential associations between miRNAs and diseases still remains to involve a large number of unclear and incomplete works that need to be further improved:

(1) The prediction accuracy still needs to be enhanced further;
(2) Isolated diseases and new miRNAs cannot be handled directly;
(3) Similarity construction processes are not accurate enough.

Around the above limitations, a global prediction method (MSFSP) that combined with multiple-similarities fusion (MSF) attribute was proposed, which could predict the associations between all diseases (including isolated diseases) and miRNAs (including new miRNAs) without needing negative samples. MSFSP mainly consisted of the following steps:

(1) Reconstructed disease similarity network (fused by disease semantic similarity, disease functional similarity, and disease Hamming similarity) and miRNA similarity network (fused by miRNA functional similarity, miRNA sequence similarity, and miRNA Hamming similarity);
(2) Reconstructed miRNA–disease network *via* integrated disease similarity, miRNA similarity, and verified Boolean network of miRNA–disease associations;
(3) Obtained the final prediction scores of miRNA–disease associations by using the space projections of reconstructed miRNA–disease network on similarities spaces.

## Materials and Methods

### Known MiRNA–Disease Associations

The experimentally verified miRNA–disease associations downloaded from HMDD v2.0 (Li Y. et al., 2014) with pre-treatment were composed of 495 processed miRNAs (formed a collection of miRNAs *M* = {*m*_1_, *m*_2_, …, *m*_*i*_, …, *m*_*n*_*m*__}, *n*_*m*_=495), 383 diseases (formed a collection of diseases *D* = {*d*_1_, *d*_2_, …*d*_*j*_, …*d*_*n*_*d*__}, *n*_*d*_=383), and 5,430 known miRNA–disease associations (formed a matrix $\text{M}{\text{D}}^{n_{m}\times n_{d}}$). The element value of **MD**(*i, j*) in $\text{M}{\text{D}}^{n_{m}\times n_{d}}$ is set to 1 if the miRNA node ***m***_***i***_ (*i* = 1, 2, ⋯*n*_*m*_) is associated with the disease node ***d***_***j***_ (*j* = 1, 2, …*n*_*d*_); otherwise, it is set to 0.

### Disease Semantic Similarity and Disease Functional Similarity

According to the description in Wang et al. (2010), disease similarities based on semantic information were denoted by matrix ${\text{D}\text{D}}_{ss}^{n_{d}\times n_{d}}$; it can be calculated *via* utilizing the arborescence attribute of disease in the MeSH database (Lowe and Barnett, 1994) where every disease node was marked in directed acyclic graph. Two diseases have more similar phenotypes when they associate with the same genes, based on which many researchers used the disease–gene associations to calculate disease functional similarity (Luo et al., 2017b; Jiang et al., 2018). As described in detail in Jiang et al. (2018), disease functional similarities were denoted by the matrix ${\text{D}\text{D}}_{fs}^{n_{d}\times n_{d}}$.

### MiRNA Functional Similarity and MiRNA Sequence Similarity

miRNA–miRNA functional similarities were downloaded from Wang et al. (2010), and the pairwise miRNA functional similarities were denoted by the matrix ${\text{M}\text{M}}_{fs}^{n_{m}\times n_{m}}$. The miRNA sequence similarities obtained from the miRBase database (Kozomara and Griffiths-Jones, 2013) were denoted by the matrix ${\text{M}\text{M}}_{ss}^{n_{m}\times n_{m}}$.

### Hamming Similarity

Hamming similarity for vectors is a function that measures the number of equal components, divided by the length of vectors (Charikar, 2002). It is known that diseases with similar phenotypes are often related to similar miRNAs. Thereby, we defined disease Hamming similarity (denoted by the matrix ${\text{D}\text{D}}_{hs}^{n_{d}\times n_{d}}$), whose element value is shown as follows:

(1) $$\begin{array}{l} \text{D}{\text{D}}_{hs}\left(i,j\right)=1\text{-}\frac{\sum_{k=1}^{n_{m}}IdSim\left(\text{M}\text{D}\left(k,i\right),\text{M}\text{D}\left(k,j\right)\right)}{n_{m}} \end{array}$$

(2) $$\begin{array}{l} IdSim\left(\text{M}\text{D}\left(k,i\right),\text{M}\text{D}\left(k,j\right)\right)=\{\begin{array}{c} 1,\text{if }\text{M}\text{D}\left(k,i\right)\neq \text{M}\text{D}\left(k,j\right)\\ 0,\text{if }\text{M}\text{D}\left(k,i\right)\text{=}\text{M}\text{D}\left(k,j\right) \end{array} \end{array}$$

where **DD**_*hs*_(*i, j*) represents the Hamming similarity between disease node *d*_*i*_ and *d*_*j*_.

Similarly, we used **MD**^*T*^ that denoted the transposed matrix of **MD** to define miRNA Hamming similarity (denoted by matrix ${\text{M}\text{M}}_{hs}^{n_{m}\times n_{m}}$). The corresponding element value in ${\text{M}\text{M}}_{hs}^{n_{m}\times n_{m}}$ is shown as follows:

(3) $$\begin{array}{l} \text{M}{\text{M}}_{hs}\left(i,j\right)=1\text{-}\frac{\sum_{k=1}^{n_{d}}IdSim\left(\text{M}{\text{D}}^{T}\left(k,i\right),\text{M}{\text{D}}^{T}\left(k,j\right)\right)}{n_{d}} \end{array}$$

(4) $$\begin{array}{l} IdSim\left(\text{M}{\text{D}}^{T}\left(k,i\right),\text{M}{\text{D}}^{T}\left(k,j\right)\right)=\{\begin{array}{c} 1,\text{ if }\text{M}{\text{D}}^{T}\left(k,i\right)\neq \text{M}{\text{D}}^{T}\left(k,j\right)\\ 0,\text{ if }\text{M}{\text{D}}^{T}\left(k,i\right)\text{=}\text{M}{\text{D}}^{T}\left(k,j\right) \end{array} \end{array}$$

where **MM**_*hs*_(*i, j*) represents the Hamming similarity between miRNA nodes *m*_*i*_ and *m*_*j*_.

### Multiple-Similarities Fusion

In this section, we used similarity kernel fusion (Wang et al., 2014; Jiang et al., 2018, 2019) to integrate three miRNA similarities (miRNA functional similarities ${\text{M}\text{M}}_{fs}^{n_{m}\times n_{m}}$, miRNA sequence similarities ${\text{M}\text{M}}_{ss}^{n_{m}\times n_{m}}$, and miRNA Hamming similarities ${\text{M}\text{M}}_{hs}^{n_{m}\times n_{m}}$) into one matrix ${\text{M}\text{M}}_{is^{*}}^{n_{m}\times n_{m}}$ that represented integrated miRNA similarities and three disease similarities (disease functional similarities ${\text{D}\text{D}}_{fs}^{n_{d}\times n_{d}}$, disease semantic similarities ${\text{D}\text{D}}_{ss}^{n_{d}\times n_{d}}$, and disease Hamming similarities ${\text{D}\text{D}}_{hs}^{n_{d}\times n_{d}}$) into one matrix ${\text{D}\text{D}}_{is^{*}}^{n_{d}\times n_{d}}$ that represented integrated disease similarities. Details on the integration are in the following discussion.

Firstly, using similar methods mentioned in Jiang et al. (2018, 2019), the corresponding sparse matrices for three miRNA similarities denoted by ${\text{M}\text{M}}_{sfs}^{n_{m}\times n_{m}}$, ${\text{M}\text{M}}_{sss}^{n_{m}\times n_{m}}$, and ${\text{M}\text{M}}_{shs}^{n_{m}\times n_{m}}$, respectively, were constructed and the corresponding sparse matrices for three disease similarities were denoted by ${\text{D}\text{D}}_{sfs}^{n_{d}\times n_{d}}$, ${\text{D}\text{D}}_{sss}^{n_{d}\times n_{d}}$, and ${\text{D}\text{D}}_{shs}^{n_{d}\times n_{d}}$, respectively.

(5) $$\begin{array}{l} \text{M}{\text{M}}_{sfs}\left(i,j\right)=\{\begin{array}{ll} 0, & \text{if }m_{j}\notin N_{m_{i}}\\ \frac{\text{M}{\text{M}}_{fs}\left(i,j\right)}{\sum_{m_{k}\in N_{m_{i}}}\text{M}{\text{M}}_{fs}\left(i,k\right)}, & \text{if }m_{j}\in N_{m_{i}} \end{array} \end{array}$$

where *N*_*m*_*i*__ represents the collection of all neighbors of miRNA node *m*_*i*_, including *m*_*i*_ in the corresponding three miRNA similarities matrices (**MM**_*sfs*_, **MM**_*sss*_, and **MM**_*shs*_), and the number of *N*_*m*_*i*__ was set to 36.

Similarly, **MM**_*sss*_(*i, j*), **MM**_*shs*_(*i, j*), **DD**_*sfs*_(*i, j*), **DD**_*sss*_(*i, j*), and **DD**_*shs*_(*i, j*) were constructed by using the above representation.

Secondly, the integrated normalized matrices and sparse matrices are as follows:

(6) $$\begin{array}{l} {\left({\bar{\text{M}\text{M}}}_{fs}\right)}^{t+1}=\delta \left(\text{M}{\text{M}}_{sfs}\times \frac{{\left({\bar{\text{M}\text{M}}}_{ss}\right)}^{t}+{\left({\bar{\text{M}\text{M}}}_{hs}\right)}^{t}}{2}\times {\text{M}\text{M}}_{sfs}^{T}\right)\\ \;+\left(1-\delta \right)\frac{{\left({\bar{\text{M}\text{M}}}_{ss}\right)}^{0}+{\left({\bar{\text{M}\text{M}}}_{hs}\right)}^{0}}{2} \end{array}$$

where ${\bar{\text{M}\text{M}}}_{ss}$ and ${\bar{\text{M}\text{M}}}_{hs}$ are the normalizations for **MM**_*ss*_ and **MM**_*hs*_, respectively. ${\text{M}\text{M}}_{sfs}^{T}$ denotes the transposed matrix of **MM**_*sfs*_; ${\left({\bar{\text{M}\text{M}}}_{ss}\right)}^{t}$and ${\left({\bar{\text{M}\text{M}}}_{hs}\right)}^{t}$ are the *t*^*th*^ iteration results of ${\bar{\text{M}\text{M}}}_{ss}$ and ${\bar{\text{M}\text{M}}}_{hs}$, respectively. *t* was set to 10 and δ was set to 0.1, which are similar as those defined in Jiang et al. (2018, 2019). ${\left({\bar{\text{M}\text{M}}}_{ss}\right)}^{0}$ and ${\left({\bar{\text{M}\text{M}}}_{hs}\right)}^{0}$ represented the initial status of ${\bar{\text{M}\text{M}}}_{ss}$ and ${\bar{\text{M}\text{M}}}_{hs}$, respectively, with the detailed calculation shown as follows:

(7) $$\begin{array}{l} {\left({\bar{\text{M}\text{M}}}_{ss}\left(i,j\right)\right)}^{0}=\frac{\text{M}{\text{M}}_{ss}\left(i,j\right)}{\text{M}{\text{M}}_{fs}\left(i,j\right)+\text{M}{\text{M}}_{ss}\left(i,j\right)+\text{M}{\text{M}}_{hs}\left(i,j\right)} \end{array}$$

(8) $$\begin{array}{l} {\left({\bar{\text{M}\text{M}}}_{hs}\left(i,j\right)\right)}^{0}=\frac{\text{M}{\text{M}}_{hs}\left(i,j\right)}{\text{M}{\text{M}}_{fs}\left(i,j\right)+\text{M}{\text{M}}_{ss}\left(i,j\right)+\text{M}{\text{M}}_{hs}\left(i,j\right)} \end{array}$$

Furthermore, similar representations of ${\left({\bar{\text{M}\text{M}}}_{ss}\right)}^{t+1}$ and ${\left({\bar{\text{M}\text{M}}}_{hs}\right)}^{t+1}$ could be obtained as that of ${\left({\bar{\text{M}\text{M}}}_{fs}\right)}^{t+1}$. After *t* + 1 iterations, the temporarily integrated miRNA similarity denoted by matrix **MM**_*is*_ was calculated as follows:

(9) $$\begin{array}{l} \text{M}{\text{M}}_{is}=\frac{{\left({\bar{\text{M}\text{M}}}_{fs}\right)}^{t+1}+{\left({\bar{\text{M}\text{M}}}_{ss}\right)}^{t+1}+{\left({\bar{\text{M}\text{M}}}_{hs}\right)}^{t+1}}{3} \end{array}$$

Thirdly, a weighted matrix ${\text{W}}_{m}^{n_{m}\times n_{m}}$ for eliminating noises during the calculation process was constructed, as mentioned in Jiang et al. (2019). Then, the finally integrated miRNA similarity denoted by matrix ${\text{M}\text{M}}_{is^{*}}^{n_{m}\times n_{m}}$ was obtained *via* taking a dot product:

(10) $$\begin{array}{l} {\text{W}}_{m}\left(i,j\right)=\{\begin{array}{cc} 1, & \text{if }m_{i}\in N_{m_{j}}\text{and }m_{j}\in N_{m_{i}}\;\\ 0, & \text{if }m_{i}\notin N_{m_{j}}\text{and }m_{j}\notin N_{m_{i}}\\ 0.5, & \text{otherwise} \end{array} \end{array}$$

(11) $$\begin{array}{l} \text{M}{\text{M}}_{is^{*}}=\text{M}{\text{M}}_{is}○{\text{W}}_{m} \end{array}$$

The finally integrated disease similarity matrix ${\text{D}\text{D}}_{is^{*}}^{n_{d}\times n_{d}}$, the weighted matrix ${\text{W}}_{d}^{n_{d}\times n_{d}}$, and the temporarily integrated disease similarity matrix ${\text{D}\text{D}}_{is}^{n_{d}\times n_{d}}$ can be calculated by a similar calculation process as that of $\text{M}{\text{M}}_{is^{*}}$:

(12) $$\begin{array}{l} \text{D}{\text{D}}_{is^{*}}=\text{D}{\text{D}}_{is}○{\text{W}}_{d} \end{array}$$

### Weighted Network Construction

On account of the hypothesis that miRNAs with similar functions are often related to the diseases with similar phenotypes, many methods for miRNA–disease association prediction were proposed. Though the network **MD** of known experimentally verified miRNA–disease association plays a very important role in these prediction methods, network **MD** is only a Boolean network which can indicate if the miRNA–disease association exits or not, without any information of the extent of association. Therefore, in order to enhance the predictive validity, we used **MD** and similarities between miRNAs (diseases) to accurately construct a weighted network with which to uncover potential miRNA–disease associations.

#### Weighted Network Construction Based on MiRNA Similarities

The contribution value of the other miRNA node *m*_*k*_(*k* ≠ *i*) to *m*_*i*_ (denoted by *C*_*m*_*k*__) was defined as follows:

(13) $$\begin{array}{l} C_{m_{k}}=\text{M}{\text{M}}_{is^{*}}\left(i,k\right)\times \text{M}\text{D}\left(k,j\right) \end{array}$$

where $\text{M}{\text{M}}_{is^{*}}\left(i,k\right)$ is the finally integrated miRNA similarity between *m*_*i*_ and *m*_*k*_, and **MD**(*k, j*) represents the Boolean value of the association between *m*_*k*_ and *d*_*j*_.

If there is an association between *m*_*k*_ and *d*_*j*_, the more similar *m*_*k*_ and *m*_*i*_ are, the higher the contribution value of *m*_*k*_ to the weight between *m*_*i*_ and *d*_*j*_. Based on the discussion above, the miRNA–disease weighted network based on miRNA similarities (denoted by ${\text{M}\text{D}}_{m}^{n_{m}\times n_{d}}$) was defined as follows:

(14) $$\begin{array}{l} \text{M}{\text{D}}_{m}\left(i,j\right)=\text{M}\text{D}\left(i,j\right)+\alpha \sum_{k=1,k\neq i}^{n_{m}}C_{m_{k}} \end{array}$$

where **MD**_*m*_(*i, j*) is the weight between miRNA node *m*_*i*_ and disease node *d*_*j*_, the equilibrium parameter being α ∈ [ 0, 1].

#### Weighted Network Construction Based on Disease Similarities

Similarly, the contribution value of the other disease nodes *d*_*k*_(*k* ≠ *i*) to *d*_*i*_ (denoted by *C*_*d*_*k*__) was defined as follows:

(15) $$\begin{array}{l} C_{d_{k}}=\text{M}\text{D}\left(i,k\right)\times \text{D}{\text{D}}_{is*}\left(k,j\right) \end{array}$$

miRNA–disease weighted network based on disease similarities (denoted by ${\text{M}\text{D}}_{d}^{n_{m}\times n_{d}}$) was defined as follows:

(16) $$\begin{array}{l} \text{M}{\text{D}}_{d}\left(i,j\right)=\text{M}\text{D}\left(i,j\right)+\beta \sum_{k=1,k\neq j}^{n_{d}}C_{d_{k}} \end{array}$$

where equilibrium parameter β ϵ [0,1].

### Space Projection Scores Based on Similarities

To enhance the predictive accuracy further, we integrated ${\text{M}\text{D}}_{d}^{n_{m}\times n_{d}}$ and ${\text{M}\text{M}}_{is^{*}}^{n_{m}\times n_{m}}$ to construct miRNA space projection scores denoted by matrix ${\text{F}}_{pm}^{n_{d}\times n_{m}}$, shown as follows:

(17) $$\begin{array}{l} {\text{F}}_{pm}\left(i,j\right)=\frac{{\text{M}\text{D}}_{d}^{T}\left(i,:\right)\times \text{M}{\text{M}}_{is*}\left(:,j\right)}{\left\|\text{M}{\text{M}}_{is*}\left(:,j\right)\right\|} \end{array}$$

where ${\text{M}\text{D}}_{d}^{T}$ is the transposed matrix of **MD**_*d*_, and ||**MM**_is*_(:, *j*)|| is the norm of vector **MM**_is*_(:, *j*).

Similarly, we integrated ${\text{M}\text{D}}_{m}^{n_{m}\times n_{d}}$ and ${\text{D}\text{D}}_{is^{*}}^{n_{d}\times n_{d}}$ to construct disease space projection scores denoted by matrix ${\text{F}}_{pd}^{n_{m}\times n_{d}}$, shown as follows:

(18) $$\begin{array}{l} {\text{F}}_{pd}\left(i,j\right)=\frac{\text{M}{\text{D}}_{m}\left(i,:\right)\times \text{D}{\text{D}}_{is^{*}}\left(:,j\right)}{\left\|\text{D}{\text{D}}_{is^{*}}\left(:,j\right)\right\|} \end{array}$$

where $\left||\text{D}{\text{D}}_{is^{*}}\left(:,j\right)|\right|$ is the norm of vector $\text{D}{\text{D}}_{is^{*}}\left(:,j\right)$.

Finally, we integrated **F**_*pm*_(*i, j*) and **F**_*pd*_(*i, j*) to obtain the final prediction score **F**_*pf*_(*i, j*), shown as follows:

(19) $$\begin{array}{l} {\text{F}}_{pf}\left(i,j\right)=\left(1-\gamma \right){\text{F}}_{pm}^{T}\left(i,j\right)+\gamma {\text{F}}_{pd}\left(i,j\right) \end{array}$$

where ${\text{F}}_{pm}^{T}$ is the transposed of matrix of **F**_*pm*_, and the equilibrium parameter γ ∈ [0, 1] represents the importance degree of **F**_*pm*_(*i, j*) and **F**_*pd*_(*i, j*).

**Figure 1:**
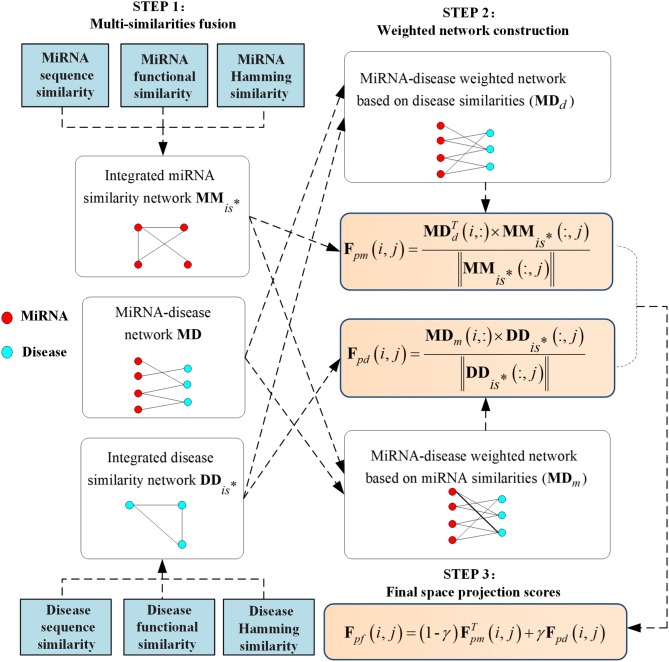
Flowchart of the whole modeling procedure.

Therefore, we will integrate disease similarities, miRNA similarities, and weighted networks to obtain the final prediction scores ${\text{F}}_{pf}^{n_{m}\times n_{d}}$, whose higher value means a higher probability that miRNA *m*_*i*_ associates with disease *d*_*j*_. The detailed calculation steps of **F**_*pf*_ are shown in Figure 1 for clarity.

## Results

### Influence of Parameter Selection on Performance

This section mainly discussed the influences of different types of parameters (weighting parameter α, β and equilibrium parameter γ) on the predictive performance of MSFSP. For simplicity, we set α and β to be of the same value.

**Figure 2:**
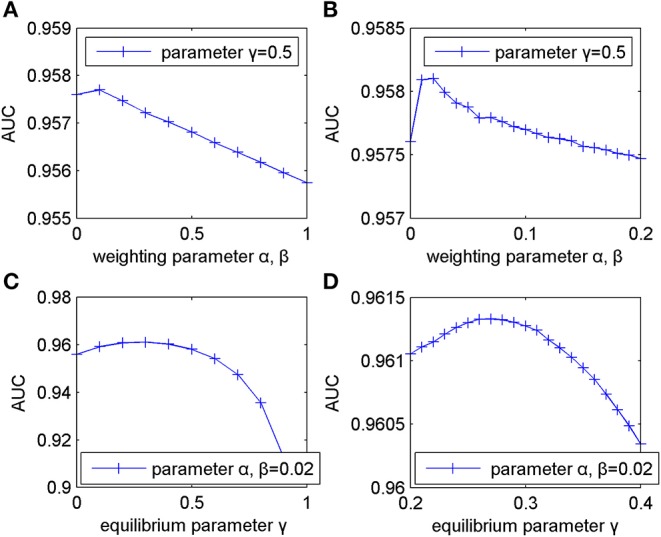
Influence of parameter variation on model predictive accuracy. **(A)** Changing weighting parameters from 0 to 1 with step-size of 0.1. **(B)** Changing weighting parameters from 0 to 0.2 with step-size of 0.01. **(C)** Changing equilibrium parameter from 0 to 1 with step-size of 0.1. **(D)** Changing equilibrium parameter from 0.2 to 0.4 with step-size of 0.01.

Firstly, we fixed γ to 0.5 and changed α and β from 0 to 1 with a step-size of 0.1. After performing LOOCV, the results showed that AUC reached an optimal value of 0.9577 when α and β were set to 0.1. Then, the AUC values decreased gradually when α and β increased from 0.1 to 1, which caused the corresponding curve to decline linearly (shown in Figure 2). Therefore, α and β should range from 0 to 0.2 to get the optimal value.

Next, in order to get more accurate weighting parameters, we fixed γ to 0.5 again and changed α and β from 0 to 0.2 with a step-size of 0.01. The corresponding changing curve is shown in Figure 2, where the optimal AUC of 0.9581 was obtained when α and β were both 0.02.

Then, based on α = β = 0.02, we evaluated the influence of γ on MSFSP in a similar way as detailed above. We increased γ from 0 to 1 with a step-size of 0.1 to obtain the corresponding results shown in Figure 2, where the optimal, suboptimal, and third-best value of AUC were obtained when γ was 0.3, 0.2, and 0.4, respectively. However, AUC decreased when γ increased from 0.4. Therefore, γ should range from 0.2 to 0.4 to get the optimal value. We increased γ from 0.2 to 0.4 with a step-size of 0.01 to get more accurate parameter values with α and β fixed to 0.02. The changing curve in Figure 2 shows the optimal value of 0.9613 when γ was 0.27.

In conclusion, our parameter selections were α = β = 0.02 and γ = 0.27.

### Comparison of Predictive Performance Under Different Situations

**Figure 3:**
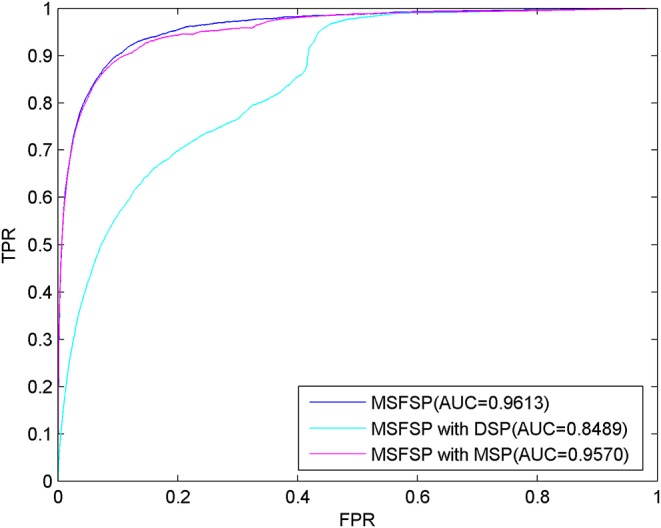
Receiver operating characteristic curves and area under the curve values *via* leave-one-out cross-validation in different situations.

We performed LOOCV to evaluate the predictive performance of MSFSP under the following different situations: (1) with all relevant information (MSFSP with all), (2) only with miRNA space projection (MSFSP with MSP), and (3) only with disease space projection (MSFSP with DSP). The ROC curves for the above different situations are shown in Figure 3, where the AUC value of MSFSP withr all was 0.9613, the AUC value of MSFSP with MSP was 0.9570, and the AUC value of MSFSP with DSP was 0.8489. Therefore, MSFSP showed reliable predictive performance for inferring miRNA–disease associations effectively.

### Comparison of Predictive Performance With Different Integrated Similarity Constructions

**Figure 4:**
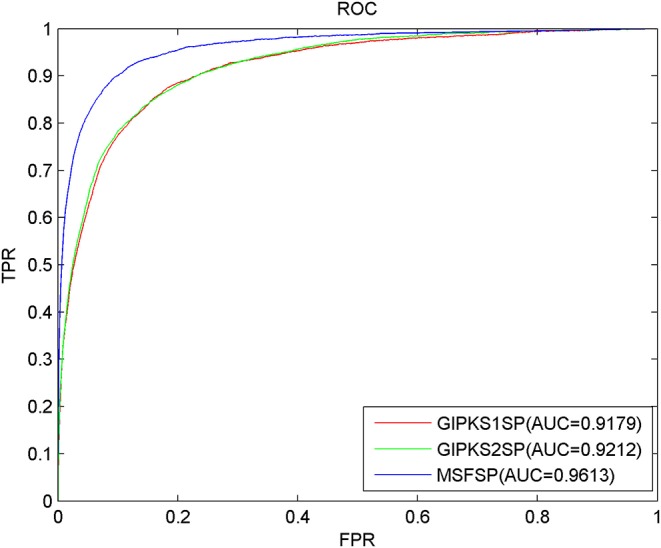
Receiver operating characteristic curves and area under the curve values of MSFSP against GIPKS1SP and GIPKS2SP.

Concerning the limitations of the sparsity and incompleteness existing in disease semantic similarity and miRNA functional similarity, we used MSF in MSFSP to construct the integrated disease similarity and the integrated miRNA similarity with which to solve these limitations. Some other researchers integrated disease semantic similarity (miRNA functional similarity) with Gaussian interaction profile kernel similarity to construct the integrated diseases similarity (the integrated miRNA similarity) with which to solve the same limitations (Chen et al., 2016c, 2017d; Chen and Huang, 2017; Zhao et al., 2018, 2019). In order to compare which of the two ways wherein integrated similarities were constructed has better predictive result, we compared MSF used in MSFSP with Gaussian interaction profile kernel similarity used in Chen and Huang (2017) and Zhao et al. (2019). We used the Gaussian interaction profile kernel similarity coming from Chen and Huang (2017) to replace MSF in MSFSP, and we got a new prediction model called GIPKS1SP as one object to be compared. Similarly, we used Gaussian interaction profile kernel similarity coming from Zhao et al. (2019) to replace MSF in MSFSP, and we got another new prediction model called GIPKS2SP as another object to be compared. After performing LOOCV, the AUCs of GIPKS1SP, GIPKS2SP, and MSFSP were 0.9179, 0.9212, and 0.9613, respectively (shown in Figure 4). The more reliable predictive performance obtained *via* MSFSP proved that MSF is better than the Gaussian interaction profile kernel similarity to construct the integrated similarities.

### Comparison to Other Methods

**Figure 5:**
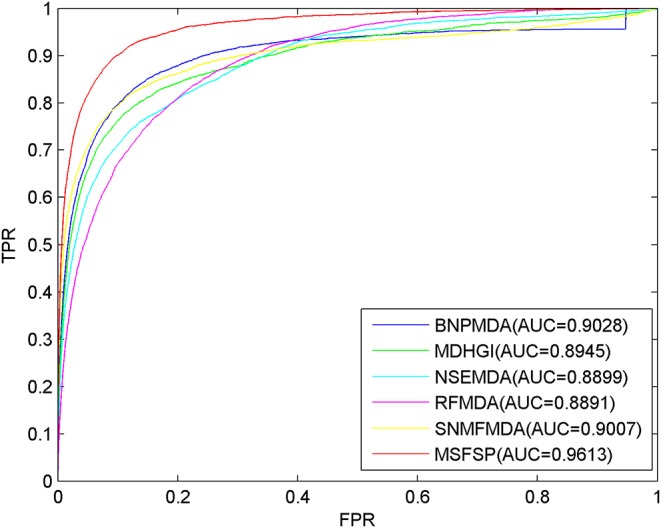
Receiver operating characteristic curves and area under the curve values of multiple-similarities fusion and space projection against other five methods.

To our knowledge, BNPMDA (Chen et al., 2018h), MDHGI (Chen et al., 2018i), NSEMDA (Wang C.-C. et al., 2019), RFMDA (Chen et al., 2018f), and SNMFMDA (Zhao et al., 2018) are the most advanced prediction methods in inferring miRNA–disease associations so far. Due to the fact that the databases used by these five methods are similar with that of MSFSP, we compared MSFSP with these five methods on the predictive performance. The LOOCV results in Figure 5 show that the AUC values of BNPMDA, MDHGI, NSEMDA, RFMDA, SNMFMDA, and MSFSP were 0.9028, 0.8945, 0.8899,0.8891, 0.9007, and 0.9613, respectively. MSFSP achieved the superior prediction effect, at 6.09, 6.94, 7.42, 7.51, and 6.30% higher than BNPMDA, MDHGI, NSEMDA, RFMDA, and SNMFMDA, respectively.

### Prediction of New MiRNAs and Isolated Diseases

With the continuously developing miRNA recognition technology, more and more miRNAs are being discovered, but whose associations with diseases are unknown. The prediction for isolated diseases and new miRNAs will definitely accelerate the scientists' understanding of the molecular mechanisms of diseases as well as how diseases occur. Therefore, the prediction for isolated diseases and new miRNAs has become a hot research topic in recent years.

**Figure 6:**
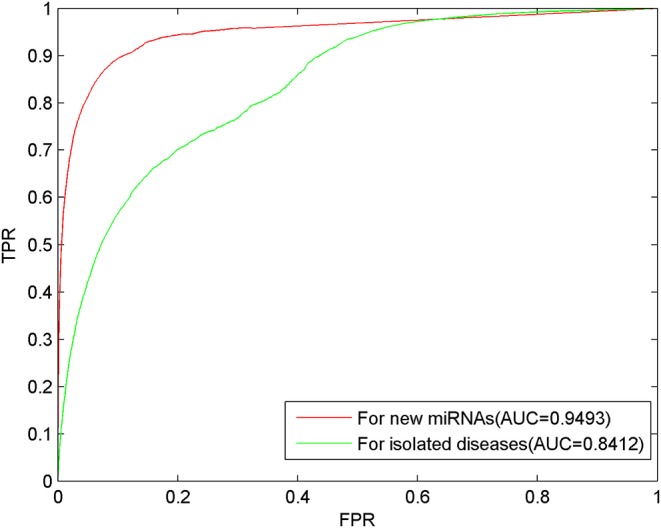
Predictions of new miRNAs and isolated diseases.

For each miRNA, we removed all related associations with diseases to simulate the new miRNA. For each disease, we removed all related associations with miRNAs to simulate the isolated disease. Through LOOCV, the prediction results shown as AUC of 0.9493 and 0.8412, respectively, were obtained, where the ROC curve demonstrated the excellent predictive performance of MSFSP on inferring new miRNA-related diseases, as well as isolated diseases related with miRNAs (as can be seen in Figure 6).

## Case Studies

To further evaluate the predictive ability of MSFSP on inferring diseases potentially related to miRNAs, we selected prostatic neoplasms and lung neoplasms as the case studies with model training and predicting on three independent databases HMDD v3.2 (Huang et al., 2018), dbDEMC 2.0 (Yang et al., 2017), and miR2Disease (Jiang et al., 2009).

### Prediction of Potential MiRNA–Disease Associations

The low detection rate of lung neoplasm, making it a common lethal disease, poses a great threat to people's lives especially in developing countries (Torre et al., 2016; Temraz et al., 2017). We used 132 known associations between lung neoplasms and miRNAs as training samples to predict the remaining unknown associations. We found the supporting evidences that 49 out of all the first 50 miRNAs related to lung neoplasms predicted by MSFSP were confirmed on the above-mentioned three databases (HMDD v3.2, dbDEMC 2.0, and miR2Disease), except hsa-mir-384 (as shown in Table 1). However, we found the association between hsa-mir-384 and lung neoplasms by searching the latest literature (Guo et al., 2019) whose publication date was after the last update of HMDD v3.2, which further confirmed the effectiveness of MSFSP for inferring diseases potentially related to miRNAs.

**Table 1:** Top 50 lung neoplasm-related miRNAs.

**Rank**	**MiRNA name**	**Database of evidence**	**Rank**	**MiRNA name**	**Database of evidence**
1	hsa-mir-16	HMDD, dbDEMC, miR2Disease	26	hsa-mir-668	dbDEMC
2	hsa-mir-106b	HMDD, dbDEMC	27	hsa-mir-208a	HMDD
3	hsa-mir-15a	HMDD, dbDEMC	28	hsa-mir-708	dbDEMC
4	hsa-mir-141	HMDD, dbDEMC, miR2Disease	29	hsa-mir-663b	dbDEMC
5	hsa-mir-15b	dbDEMC	30	hsa-mir-196b	HMDD, dbDEMC
6	hsa-mir-194	HMDD, dbDEMC	31	hsa-mir-328	HMDD, dbDEMC
7	hsa-mir-130a	HMDD, dbDEMC, miR2Disease	32	hsa-mir-342	HMDD, dbDEMC
8	hsa-mir-151a	dbDEMC	33	hsa-mir-149	HMDD, dbDEMC
9	hsa-mir-429	dbDEMC, miR2Disease	34	hsa-mir-1236	dbDEMC
10	hsa-mir-99a	HMDD, dbDEMC, miR2Disease	35	hsa-mir-320a	dbDEMC
11	hsa-mir-122	HMDD, dbDEMC	36	hsa-mir-370	dbDEMC
12	hsa-mir-195	HMDD, dbDEMC, miR2Disease	37	hsa-mir-181d	dbDEMC
13	hsa-mir-20b	dbDEMC	38	hsa-mir-144	HMDD, dbDEMC
14	hsa-mir-193b	dbDEMC	39	hsa-mir-302b	dbDEMC
15	hsa-mir-378a	dbDEMC	40	hsa-mir-363	dbDEMC
16	hsa-mir-129	HMDD, dbDEMC	41	hsa-mir-424	dbDEMC
17	hsa-mir-153	HMDD, dbDEMC	42	hsa-mir-130b	HMDD, dbDEMC
18	hsa-mir-451a	HMDD, dbDEMC	43	hsa-mir-373	HMDD, dbDEMC
19	hsa-mir-10a	HMDD, dbDEMC	44	hsa-mir-204	dbDEMC, miR2Disease
20	hsa-mir-28	dbDEMC	45	hsa-mir-211	dbDEMC
21	hsa-mir-92b	dbDEMC	46	hsa-mir-139	HMDD, dbDEMC, miR2Disease
22	hsa-mir-625	dbDEMC	47	hsa-mir-367	dbDEMC
23	hsa-mir-152	HMDD, dbDEMC	48	hsa-mir-384	Unconfirmed
24	hsa-mir-296	dbDEMC	49	hsa-mir-148b	HMDD, dbDEMC
25	hsa-mir-23b	dbDEMC	50	hsa-mir-423	HMDD, dbDEMC, miR2Disease

Prostatic neoplasm is a disease occurring in the male reproductive system, especially common in countries with severely aging population, but in recent years, more and more prostatic neoplasms occur in young people (Siegel et al., 2016). We used 118 known associations between prostatic neoplasms and miRNAs as training samples to predict the remaining unknown associations. A total of 48 out of the first 50 miRNAs related to prostatic neoplasms predicted by MSFSP were confirmed on relevant databases (HMDD v3.2, dbDEMC 2.0, and miR2Disease), except hsa-mir-633 and hsa-mir-300 (ranked 46th and 47th, respectively) (as shown in Table 2). Although there is no evidence that shows the association between these two miRNAs and prostatic neoplasms by now, we believe that some evidences will be found by scientists in the near future.

**Table 2:** Top 50 prostatic neoplasm-related miRNAs.

**Rank**	**MiRNA name**	**Database of evidence**	**Rank**	**MiRNA name**	**Database of evidence**
1	hsa-mir-29c	HMDD, dbDEMC	26	hsa-mir-1229	dbDEMC
2	hsa-mir-10b	dbDEMC, miR2Disease	27	hsa-mir-944	dbDEMC
3	hsa-mir-429	HMDD	28	hsa-mir-1227	HMDD, dbDEMC
4	hsa-mir-19a	HMDD, dbDEMC	29	hsa-mir-451a	dbDEMC
5	hsa-mir-155	HMDD, dbDEMC	30	hsa-mir-139	HMDD, dbDEMC
6	hsa-mir-181a	HMDD, dbDEMC, miR2Disease	31	hsa-mir-625	dbDEMC
7	hsa-mir-210	HMDD, dbDEMC, miR2Disease	32	hsa-mir-150	HMDD, dbDEMC
8	hsa-mir-199b	HMDD, dbDEMC, miR2Disease	33	hsa-mir-128	HMDD, dbDEMC
9	hsa-mir-19b	HMDD, dbDEMC, miR2Disease	34	hsa-mir-370	HMDD, dbDEMC, miR2Disease
10	hsa-mir-18a	HMDD, dbDEMC	35	hsa-mir-18b	dbDEMC
11	hsa-mir-142	dbDEMC	36	hsa-mir-28	dbDEMC
12	hsa-mir-9	HMDD, dbDEMC	37	hsa-mir-135a	HMDD, dbDEMC
13	hsa-mir-192	HMDD, dbDEMC	38	hsa-mir-10a	HMDD, dbDEMC, miR2Disease
14	hsa-mir-125a	dbDEMC, miR2Disease	39	hsa-mir-149	HMDD, dbDEMC, miR2Disease
15	hsa-let-7f	dbDEMC, miR2Disease	40	hsa-mir-140	dbDEMC
16	hsa-mir-24	HMDD, dbDEMC, miR2Disease	41	hsa-mir-20b	HMDD, dbDEMC
17	hsa-let-7i	dbDEMC	42	hsa-mir-302b	dbDEMC
18	hsa-let-7e	dbDEMC	43	hsa-mir-328	dbDEMC
19	hsa-let-7g	HMDD, dbDEMC, miR2Disease	44	hsa-mir-30e	dbDEMC
20	hsa-mir-7	HMDD, dbDEMC	45	hsa-mir-103b	dbDEMC
21	hsa-mir-196a	HMDD, dbDEMC	46	hsa-mir-633	Unconfirmed
22	hsa-mir-206	HMDD, dbDEMC	47	hsa-mir-300	Unconfirmed
23	hsa-mir-138	HMDD	48	hsa-mir-30b	dbDEMC, miR2Disease
24	hsa-mir-30a	HMDD, dbDEMC, miR2Disease	49	hsa-mir-497	HMDD, dbDEMC, miR2Disease
25	hsa-mir-103a	dbDEMC	50	hsa-mir-663b	dbDEMC

### Prediction of Isolated Disease-Related MiRNAs

To further evaluate the predictive performance of MSFSP for isolated diseases which are those without any known associations, we removed all 118 known associations related to prostatic neoplasms to simulate the isolated disease condition. The supporting evidences for the top 50 prostatic neoplasm-related miRNAs predicted were all found from the relevant databases (HMDD v3.2, dbDEMC 2.0, and miR2Disease) (as shown in Table 3). Similarly, we removed all 132 known associations related to lung neoplasms to simulate the isolated disease condition. The supporting evidences on the top 50 lung neoplasm-related miRNAs predicted were all found from the above-mentioned three relevant databases (as shown in Table 4). The supporting evidences confirmed that the predictive accuracy for the above two simulated objects were both 100%, which further showed the excellent predictive performance of MSFSP on inferring diseases potentially related to miRNAs and isolated diseases related to miRNAs.

**Table 3:** Top 50 isolated disease-related miRNAs (prostatic neoplasm as a case).

**Rank**	**MiRNA name**	**Database of evidence**	**Rank**	**MiRNA name**	**Database of evidence**
1	hsa-mir-125b	HMDD, dbDEMC, miR2Disease	26	hsa-let-7a	HMDD, dbDEMC, miR2Disease
2	hsa-mir-21	HMDD, dbDEMC, miR2Disease	27	hsa-mir-92a	HMDD
3	hsa-mir-145	HMDD, dbDEMC, miR2Disease	28	hsa-mir-143	HMDD, miR2Disease
4	hsa-mir-99a	HMDD, dbDEMC, miR2Disease	29	hsa-mir-133b	HMDD, dbDEMC
5	hsa-mir-200c	HMDD, dbDEMC	30	hsa-mir-18a	HMDD, dbDEMC
6	hsa-mir-155	HMDD, dbDEMC	31	hsa-mir-146b	HMDD, dbDEMC
7	hsa-mir-141	HMDD, dbDEMC, miR2Disease	32	hsa-let-7g	HMDD, dbDEMC, miR2Disease
8	hsa-mir-200a	HMDD, dbDEMC	33	hsa-mir-218	HMDD, dbDEMC, miR2Disease
9	hsa-mir-183	HMDD, dbDEMC, miR2Disease	34	hsa-let-7c	HMDD, dbDEMC, miR2Disease
10	hsa-mir-100	HMDD, dbDEMC, miR2Disease	35	hsa-let-7i	dbDEMC
11	hsa-mir-9	dbDEMC	36	hsa-let-7f	dbDEMC, miR2Disease
12	hsa-mir-199a	HMDD, dbDEMC, miR2Disease	37	hsa-let-7d	HMDD, dbDEMC, miR2Disease
13	hsa-mir-34c	HMDD, dbDEMC	38	hsa-mir-7	HMDD, dbDEMC
14	hsa-mir-126	HMDD, dbDEMC, miR2Disease	39	hsa-mir-203	HMDD, dbDEMC
15	hsa-mir-29c	HMDD, dbDEMC	40	hsa-mir-1	HMDD, dbDEMC
16	hsa-mir-20a	HMDD, dbDEMC, miR2Disease	41	hsa-mir-574	HMDD, dbDEMC
17	hsa-mir-17	HMDD, dbDEMC, miR2Disease	42	hsa-let-7e	dbDEMC
18	hsa-mir-19a	HMDD, dbDEMC	43	hsa-mir-34b	HMDD, dbDEMC
19	hsa-mir-146a	HMDD, dbDEMC, miR2Disease	44	hsa-mir-101	HMDD, dbDEMC, miR2Disease
20	hsa-mir-200b	HMDD, dbDEMC	45	hsa-mir-19b	HMDD, dbDEMC, miR2Disease
21	hsa-mir-27a	HMDD, dbDEMC, miR2Disease	46	hsa-mir-10b	dbDEMC, miR2Disease
22	hsa-mir-34a	HMDD, dbDEMC, miR2Disease	47	hsa-mir-375	HMDD, dbDEMC, miR2Disease
23	hsa-let-7b	HMDD, dbDEMC, miR2Disease	48	hsa-mir-182	HMDD, dbDEMC, miR2Disease
24	hsa-mir-429	HMDD	49	hsa-mir-221	HMDD, dbDEMC, miR2Disease
25	hsa-mir-205	HMDD, miR2Disease	50	hsa-mir-142	dbDEMC

**Table 4:** Top 50 isolated disease-related miRNAs (lung neoplasm as a case).

**Rank**	**MiRNA name**	**Database of evidence**	**Rank**	**MiRNA name**	**Database of evidence**
1	hsa-mir-21	HMDD, dbDEMC, miR2Disease	26	hsa-let-7g	HMDD, dbDEMC, miR2Disease
2	hsa-mir-125b	HMDD, dbDEMC, miR2Disease	27	hsa-mir-148a	HMDD, dbDEMC, miR2Disease
3	hsa-mir-155	HMDD, dbDEMC, miR2Disease	28	hsa-let-7d	HMDD, dbDEMC, miR2Disease
4	hsa-mir-34a	HMDD, dbDEMC	29	hsa-mir-101	HMDD, dbDEMC, miR2Disease
5	hsa-mir-375	HMDD, dbDEMC	30	hsa-mir-205	HMDD, dbDEMC, miR2Disease
6	hsa-mir-146a	HMDD, dbDEMC, miR2Disease	31	hsa-let-7e	HMDD, dbDEMC, miR2Disease
7	hsa-mir-1	HMDD, dbDEMC	32	hsa-mir-93	HMDD, dbDEMC, miR2Disease
8	hsa-mir-31	HMDD, dbDEMC, miR2Disease	33	hsa-mir-143	HMDD, dbDEMC, miR2Disease
9	hsa-mir-34c	HMDD, dbDEMC	34	hsa-mir-17	HMDD, dbDEMC, miR2Disease
10	hsa-mir-145	HMDD, dbDEMC, miR2Disease	35	hsa-mir-183	HMDD, dbDEMC, miR2Disease
11	hsa-let-7a	HMDD, dbDEMC, miR2Disease	36	hsa-mir-20a	HMDD, dbDEMC, miR2Disease
12	hsa-mir-221	HMDD, dbDEMC, miR2Disease	37	hsa-mir-200b	HMDD, dbDEMC, miR2Disease
13	hsa-mir-486	HMDD, dbDEMC	38	hsa-mir-133a	HMDD, dbDEMC
14	hsa-mir-100	HMDD, dbDEMC	39	hsa-mir-193b	dbDEMC
15	hsa-mir-16	HMDD, dbDEMC, miR2Disease	40	hsa-mir-27a	HMDD, dbDEMC
16	hsa-mir-126	HMDD, dbDEMC, miR2Disease	41	hsa-let-7c	HMDD, dbDEMC, miR2Disease
17	hsa-let-7b	HMDD, dbDEMC, miR2Disease	42	hsa-mir-196a	HMDD, dbDEMC
18	hsa-mir-200c	HMDD, dbDEMC, miR2Disease	43	hsa-mir-9	HMDD, dbDEMC, miR2Disease
19	hsa-mir-34b	HMDD, dbDEMC	44	hsa-mir-29c	HMDD, dbDEMC, miR2Disease
20	hsa-mir-7	HMDD, dbDEMC, miR2Disease	45	hsa-mir-218	HMDD, dbDEMC, miR2Disease
21	hsa-mir-146b	HMDD, dbDEMC, miR2Disease	46	hsa-mir-130a	HMDD, dbDEMC, miR2Disease
22	hsa-mir-133b	HMDD, dbDEMC, miR2Disease	47	hsa-mir-222	HMDD, dbDEMC
23	hsa-mir-223	HMDD, dbDEMC	48	hsa-mir-15a	HMDD, dbDEMC
24	hsa-mir-199a	HMDD, dbDEMC, miR2Disease	49	hsa-mir-19a	HMDD, dbDEMC, miR2Disease
25	hsa-mir-499a	HMDD, dbDEMC	50	hsa-mir-141	HMDD, dbDEMC, miR2Disease

## Discussion and Conclusion

Considering that the identification of complex disease-related miRNAs is still a key research topic in the bio-medical field, we proposed a computational model called MSFSP that made the following contributions for the identification of miRNA–disease associations: (1) Compared to other methods, MSFSP can enhance the predictive accuracy effectively with an AUC value of 0.9613, which is higher than those of the other current classical computational models; (2) MSFSP implements prediction without needing negative samples; (3) MSFSP solved the inherent limitations of sparsity and incompleteness existing in current datasets *via* multiple similarities fusion; (4) MSFSP can be used to infer new miRNAs and isolated diseases, with AUC values of 0.9493 and 0.8412, respectively; (5) The predicted top 50 results for prostatic neoplasms and lung neoplasms as two cases agree well with the supporting evidences found in HMDD v3.2, dbDEMC 2.0, and miR2Disease, with the consistency of 98 and 96% respectively; (6) The predicted top 50 results for the isolated diseases simulated agree well with the supporting evidences found in HMDD v3.2, dbDEMC 2.0, and miR2Disease, with the consistency of 100% for both.

The reliable performance of MSFSP achieved can be attributed to the following factors: (1) Different biological information data were fused in MSFSP to construct the integrated miRNA similarity network and the integrated disease similarity network; (2) More accurate miRNA–disease correlations were described by weighted networks that were integrated with the disease similarity network, the miRNA similarity network, and the experimentally verified Boolean network of miRNA–disease associations; (3) MiRNA space projection scores and disease space projection scores were combined to obtain the final prediction scores, which avoided the invalid inference for new miRNAs only with disease space projection scores and the invalid inference for isolated diseases only with miRNA space projection scores.

MSFSP still has some limitations which need to be improved in the future besides its excellent prediction results. Firstly, during miRNA similarity and disease similarity calculation, the known miRNA–disease associations demand extra increase in some amount of overhead because the similarity calculation needs to be redone in LOOCV. Secondly, the construction of miRNA similarity network and disease similarity network is not accurate enough, although the accuracy has been somewhat enhanced by integrating various information. Furthermore, MSFSP can only predict if an association between miRNA and a disease exists or not, but not the specific regulatory mechanism.

## Data Availability Statement

All datasets generated for this study are included in the article/supplementary material.

## Author Contributions

YZ and MC conceived the concept of the work and designed the experiments and wrote the paper. MC, YZ, XC, and HW performed the literature search. MC, YZ, and XC collected and analyzed the data. All authors have approved the manuscript.

## Conflict of Interest

The authors declare that the research was conducted in the absence of any commercial or financial relationships that could be construed as a potential conflict of interest.
